# Notch Signaling as a Regulator of the Tumor Immune Response: To Target or Not To Target?

**DOI:** 10.3389/fimmu.2018.01649

**Published:** 2018-07-16

**Authors:** Mahnaz Janghorban, Li Xin, Jeffrey M. Rosen, Xiang H.-F. Zhang

**Affiliations:** ^1^Department of Molecular and Cellular Biology, Baylor College of Medicine, Houston, TX, United States; ^2^Lester and Sue Smith Breast Center, Baylor College of Medicine, Houston, TX, United States; ^3^Dan L. Duncan Cancer Center, Baylor College of Medicine, Houston, TX, United States; ^4^McNair Medical Institute, Baylor College of Medicine, Houston, TX, United States

**Keywords:** Notch, cancer stem cell, immune response, immune-suppressive microenvironment, Notch therapy

## Abstract

The Notch signaling pathway regulates important cellular processes involved in stem cell maintenance, proliferation, development, survival, and inflammation. These responses to Notch signaling involving both canonical and non-canonical pathways can be spatially and temporally variable and are highly cell-type dependent. Notch signaling can elicit opposite effects in regulating tumorigenicity (tumor-promoting versus tumor-suppressing function) as well as controlling immune cell responses. In various cancer types, Notch signaling elicits a “cancer stem cell (CSC)” phenotype that results in decreased proliferation, but resistance to various therapies, hence potentially contributing to cell dormancy and relapse. CSCs can reshape their niche by releasing paracrine factors and inflammatory cytokines, and the niche in return can support their quiescence and resistance to therapies as well as the immune response. Moreover, Notch signaling is one of the key regulators of hematopoiesis, immune cell differentiation, and inflammation and is implicated in various autoimmune diseases, carcinogenesis (leukemia), and tumor-induced immunosuppression. Notch can control the fate of various T cell types, including Th1, Th2, and the regulatory T cells (Tregs), and myeloid cells including macrophages, dendritic cells, and myeloid-derived suppressor cells (MDSCs). Both MDSCs and Tregs play an important role in supporting tumor cells (and CSCs) and in evading the immune response. In this review, we will discuss how Notch signaling regulates multiple aspects of the tumor-promoting environment by elucidating its role in CSCs, hematopoiesis, normal immune cell differentiation, and subsequently in tumor-supporting immunogenicity.

## Introduction

**Figure 1:**
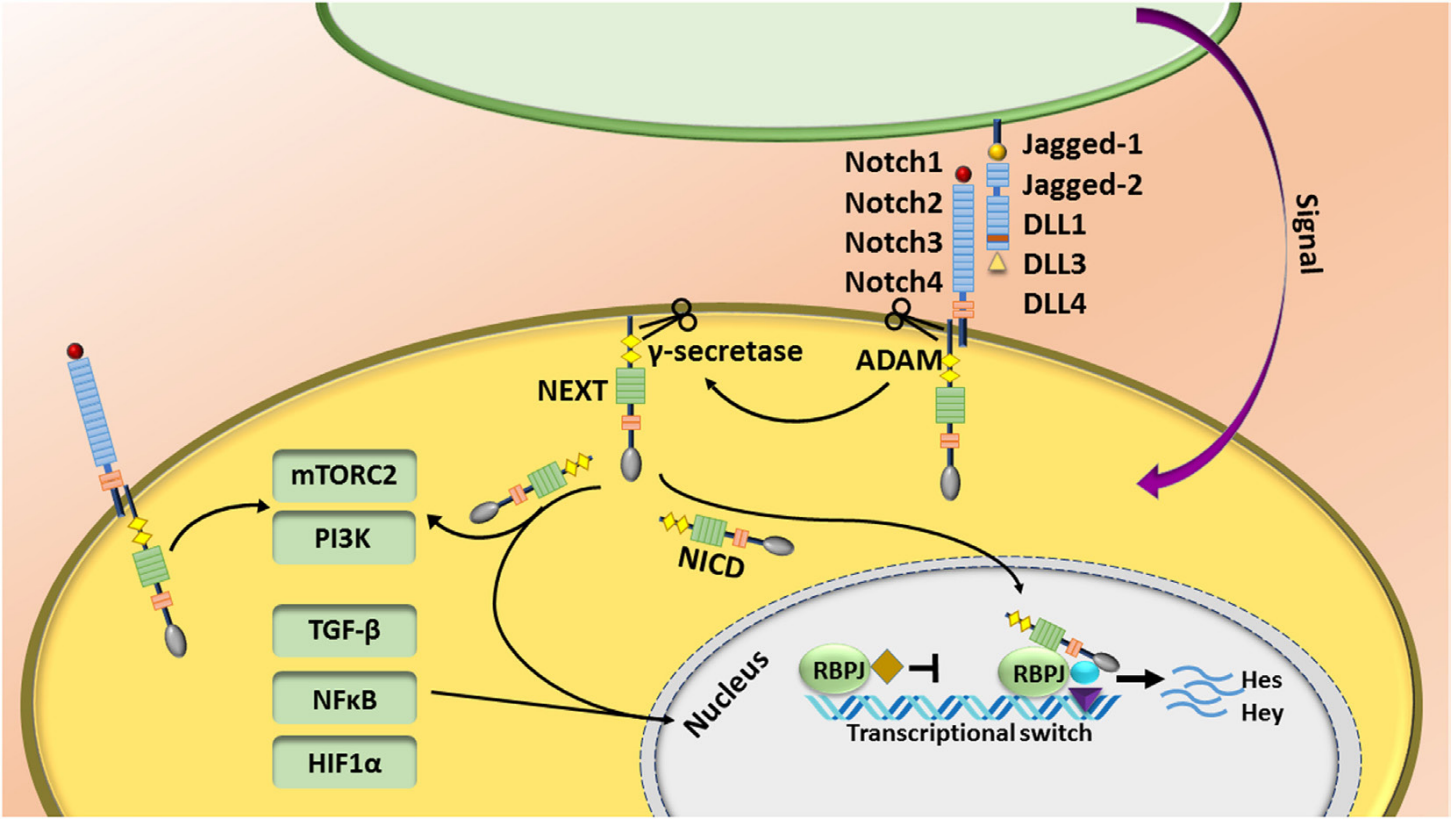
The Notch pathway is regulated by short-range cell–cell signaling. Notch is activated by interaction of one of the Notch receptors with its ligands and induces a sequence of proteolytic event leading to production of the Notch intracellular domain (NICD). NICD translocates to the nucleus, binds to the RBPJ complex, and recruits other transcriptional co-activators to initiate the transcription of target genes. Notch also regulates other target genes controlled by the TGF-β, NFκB, mTORC2, and HIF1α pathways.

The Notch pathway is regulated by short-range cell–cell signaling activated by interaction of one of the Notch receptors (Notch1–4) with different types of “canonical” ligands (Jagged1, Jagged2, DLL1, DLL3, or DLL4) [reviewed in Ref. (1)] or non-canonically through activation of other pathways such as NFκB, Wnt, TGF-β, and STAT3 [reviewed in detail elsewhere (2–4)]. The canonical Notch pathway is activated by a sequence of proteolytic events following binding of the ligand to the Notch receptor. First, the Notch receptor is cleaved by ADAM metalloproteases at the S2 site, generating a membrane-anchored Notch extracellular truncation fragment, which is further cleaved by the γ-secretase complex at S3 and S4 sites (1). Following γ-secretase cleavage, the Notch intracellular domain (NICD) releases and translocates to the nucleus where it associates with CSL—the transcriptional repressor CBF1/suppressor of hairless/Lag-1—(or the human homolog RBPJ—recombining binding protein suppressor of hairless). This is accompanied by recruitment of many transcriptional co-activators such as mastermind like (MAML1–3) to initiate the transcription of target genes (1). Because of lack of a DNA-binding motif, Notch binds to its canonical CSL (RBPJ) complex, or other pathway co-activators/repressors. Thus, Notch can regulate other target genes controlled by the TGF-β, NFκB, mTORC2, PI3K, and HIF1α pathways in the cytoplasm and/or nucleus. Although target gene expression is cell-type and context dependent, Hes and Hey families are the most characterized target genes of Notch signaling pathway (Figure 1) (5, 6).

This review is focused on the role of Notch signaling as a regulator of the tumor immune response. We will first describe the role of Notch during normal immune cell homeostasis and activation of effector cells, and then discuss the interplay between tumor cells [cancer stem cells (CSCs)] and immune cells in the tumor microenvironment. This information will need to be taken into consideration when designing new therapeutic strategies for Notch inhibition.

## Notch in Normal Immune Cell Homeostasis

Notch signaling is one of the key regulators of hematopoiesis, immune cell differentiation, and inflammation and is implicated in various autoimmune diseases and tumor-induced immunosuppression. Notch can control the differentiation and function of both innate and adaptive immunity including dendritic cells (DCs), natural killer (NK) cells, and various T cell types [Th1, Th2, and the regulatory T cells (Tregs)].

### Normal Immune Cell Differentiation

Numerous studies have investigated the role of Notch during embryonic and adult hematopoiesis. Various Notch ligands promote self-renewal of hematopoietic stem cells (HSCs) and suppress differentiation. Notch1 expression has been identified in bone marrow progenitor cells. In addition, while Jagged-1 expression in osteoblasts correlates with increased numbers of HSCs, canonical Notch signaling seems to be dispensable for adult hematopoiesis in bone marrow (7–9). More importantly, Notch signaling plays an essential role during T cell lineage commitment. Notch acts as a checkpoint to ensure T cell lineage differentiation by opposing the commitment to other cell lineages, such as B cells, myeloid cells, and DCs. The role of Notch signaling during each stage of immune cell development has been reviewed in detail elsewhere (10).

Notch1 regulates T cell lineage commitment from the common lymphoid progenitor cells and suppresses B cell development in the bone marrow (11, 12). Notch1 knockdown completely blocks T cell development and increases the accumulation of ectopic B cells in the thymus (12, 13). Moreover, Notch1 [and maybe also Notch 2 (14)] regulates the early phases of T cell differentiation in the thymus (through DLL4), but its expression needs to be decreased before T cells can fully differentiate (15). Upon migration of immature B cells from bone marrow to spleen, an increased level of Notch2 expression regulates the maturation of a subset of B cells that reside in the marginal zone, MZB cells. However, Notch2 does not control other mature B cells including follicular B cells and plasma cells (16). Moreover, *in vitro* studies have shown that Notch signaling enhances T- and NK cell differentiation from human hematopoietic progenitor cells (CD34^+^), while inhibiting B cell differentiation (14, 17). Notch also has opposing roles in controlling cell fate decisions between two different types of NK cells, i.e., conventional NK cells versus innate lymphoid cell (ILC)-derived natural cytotoxicity receptor (NCR) NKp44^+^ group (NCR^+^ILC3)—at different maturational stages of progenitor cells. This is dependent on the type of the progenitor cells. Notch can augment the differentiation of one type of these NK cells while suppressing the other types (14).

Notch also regulates the differentiation of myeloid cells. Notch signaling (transient activity) has been shown to mediate myeloid differentiation by increasing mRNA levels of the myeloid-specific transcription factor PU.1 (18). Notch1 and Notch2 are highly expressed in monocytes and in combination with GM-CSF and TNFα skew cell fate decision of DCs over macrophages (19). DLL and Jagged ligands appear to elicit opposite effects in myeloid cells, where fibroblasts expressing DLL1 promote differentiation of DCs and activation of Notch, although Jagged-1 promotes immature myeloid cells (20). In the spleen, Notch2 (probably through DLL1, as expressed in the marginal zone) controls the survival of DCs (also identified as Cx3cr1^low^ Esam^high^ DC subset), which is required for efficient T cell priming (21). Altogether, these studies have demonstrated spatiotemporally regulated roles of Notch in immune cell differentiation.

### Effector T Cell Differentiation

During the immune response, antigen-presenting cells (APCs) activate naïve T cells and trigger their clonal cell expansion into various T helper cells dictated by different sets of signaling pathways and cytokines. Notch signaling controls many aspects of effector T cell differentiation including CD4^+^ T helper cells—Th1, Th2, Th9, and Th17—Tregs, and CD8^+^ T cells [reviewed in Ref. (22)]. Functionally, Th1 cells are required for clearance of intracellular pathogens and viruses and mediating autoimmune diseases. Th2 cells mediate immunity against helminth parasites and allergic reactions. Th17 cells are critical for controlling extracellular bacterial and fungal infections and mediating autoimmunity (22, 23). Tregs are involved in the regulation of peripheral self-tolerance and tumor immunosuppression (24).

A low level of expression of Notch1 and Notch2 has been detected in naïve CD4^+^ and CD8^+^ T cells and their expression is activated through many canonical and non-canonical mechanisms such as T cell receptor (TCR) signaling and different cytokines (22, 25). The role of Notch in regulating Th1 and Th2 differentiation versus function is somewhat controversial. Notch appears to act as an unbiased amplifier of these Th programs by sensitizing cells to their microenvironmental cues, but lacks the direct capacity of instructing specific Th differentiation (23). Notch directly regulates gene expression of master regulators of Th1: T-bet and interferon-γ (IFNγ) (23), Th2: IL4 (also in NKT cells) and GATA3 (26–29), and Th17: IL17 and Rorγt (23, 30). Therefore, depending on the strength of the upstream inflammatory signaling, Notch may serve as a hub to regulate and also synergize with key signaling pathways important for Th commitment such as mTOR–AKT and NFκB to regulate Th differentiation (22). However, alternatively, there are other studies that have shown a more direct role of Notch in the control of the types immune cell responses, e.g., both *in vitro* and *in vivo* studies have shown a greater association of DLL family ligands with the development of IFNγ-secreting Th1 cells and Th17, while Jagged family ligands elicit Th2, Th9, and Treg responses (10, 22, 27). Notch also controls the survival and maintenance of memory CD4^+^ T cells which are essential for preventing recurrent infection (31). The studies highlight the complexity of the Notch signaling pathway during immune cell response.

Regulatory T cells are an immunosuppressive subpopulation of CD4^+^ cells that express Forkhead box P3 (FoxP3) and are generated from naïve CD4^+^ T cells following stimulation with TGF-β1 (32). Tregs are involved in the regulation of peripheral self-tolerance, tissue repair, and the control of pro-inflammatory immune responses, as well as the prevention of the immune response to tumors (24). Both Jagged-1 and Jagged-2 increase the generation of Tregs, e.g., Jagged-2 expression on hematopoietic progenitor cells increases the expansion of Tregs (33). Bone marrow mesenchymal stem cells educate DCs to promote a Treg expansion *via* Jagged-1 (34). Interestingly, upon Th2 stimulation, bone marrow-derived DCs express Jagged-2 (33), which can potentially regulate Treg function. Notch-1 and TGF-β cooperatively regulate the master regulator of Tregs, Foxp3 gene expression and hence directly induce peripheral Tregs (32). Altogether, Notch signaling is important in the regulation of Tregs, which can contribute to tumor-induced immunosuppression as discussed later in this review.

CD8^+^ T cell differentiation is also regulated by Notch signaling. Naïve CD8^+^ T cells differentiate into cytotoxic T lymphocytes (CTLs) upon recognition of antigens presented by MHC class 1 APCs. CTLs exert their functions by secreting IFNγ, transporting perforins and granzymes to lyse target cells, and inducing apoptosis through FAS-FAS ligand (FASL) (10). Notch1 directly binds to the promoter of EMOES—one of the master regulators of CTL differentiation, perforin, and granzyme B—and therefore enhances CTL differentiation (35). DLL1 expressing DCs activate Notch2 in CD8^+^ T cells and promote T cell cytotoxicity by increasing the expression of granzyme B (36). Moreover, activated CD8^+^ T cells choose between short-lived terminal effector cells (TECs) or memory precursor cells (MPCs). Notch signaling controls the fate decision of TECs over MPCs (37, 38), providing more evidence illustrating the complexity of Notch regulation of different cell fate decisions and functions.

Interestingly, recent studies have discovered a new subset of DCs that express high levels of DLL4 under inflammatory conditions (39, 40) [reviewed in Ref. (41)]. Immature DCs are fully differentiated through activation of pattern-recognition receptors including toll-like receptors (TLRs). Immature DCs express low levels of DLL4 and upon activation by TLR7/8, express high levels of DLL4 (41). It seems that at some point during DC differentiation, DLL4 expression is elevated and that DLL4^+^ DCs have a greater ability than DLL4^−^ DCs to promote the generation of Th1 and Th17 T cells producing IFNγ and IL-17, respectively (39, 40). Interestingly, inhibiting DLL4 abrogates efficient effector T cell function (42, 43). DLL4^+^ DCs are also important for promoting the differentiation and expansion of CD8^+^ T cells (41). Altogether, these results show that Notch plays an important role in regulating normal immune cell differentiation and the regulation of immune cell function. The role of Notch in the tumor immune response will be discussed in more detail below.

## Notch in the Cancer Immune Response

**Figure 2:**
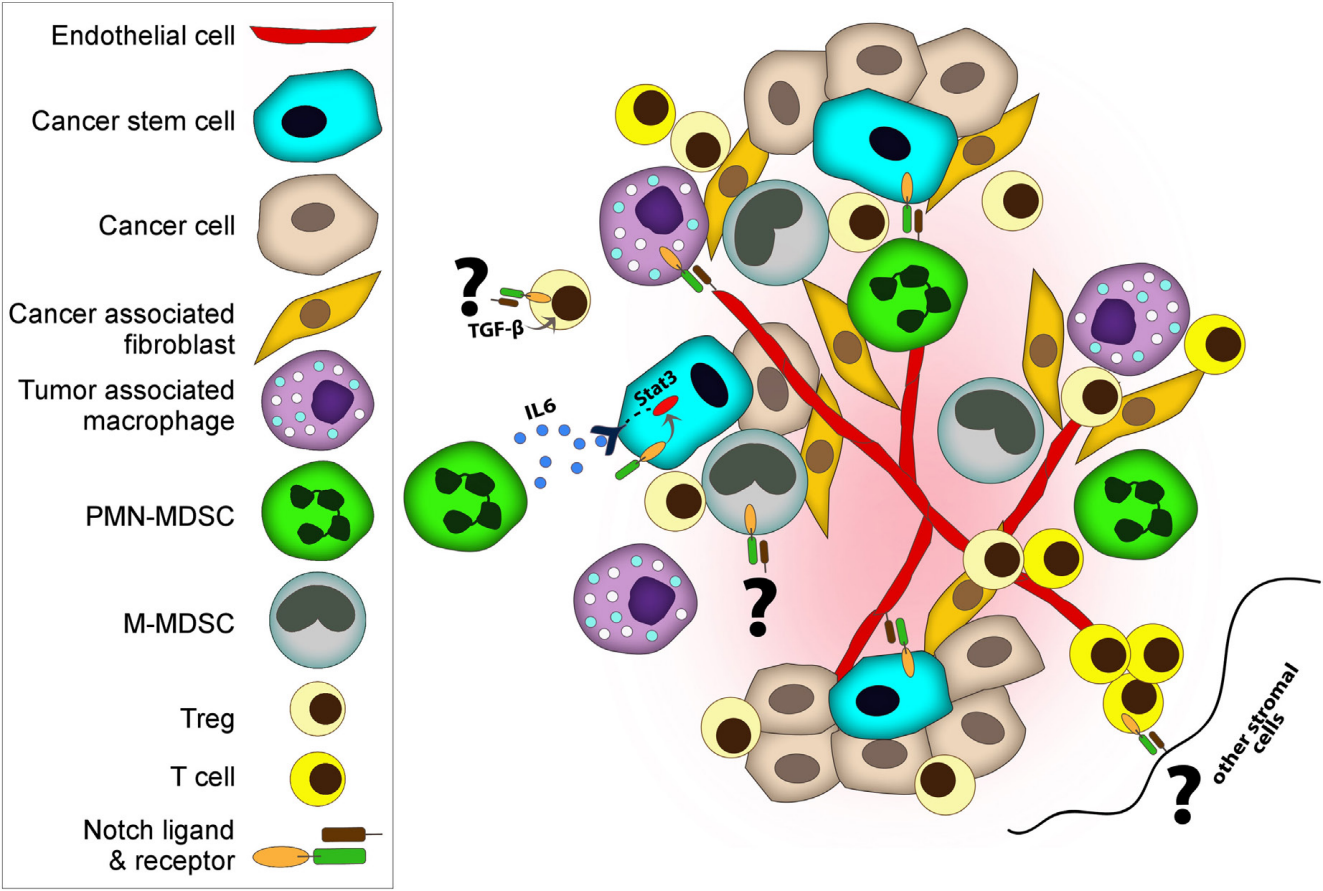
Notch regulates both cancer cells and cancer stem cells (CSCs) and many components of tumor microenvironment including immune cells, fibroblasts, and endothelial. Notch co-operates with various key signaling pathways to exert its functions. Tumor-associated macrophages and myeloid-derived suppressor cells (MDSCs) promote tumor progression by various mechanisms including suppressing immune cells, supporting CSC, and enhancing angiogenesis and metastasis. (i) Deregulated Notch activity in MDSC results in expansion of M-MDSC. (i and ii) MDSCs induce Notch signaling in cancer cells and promote CSC capacity. (iii) MDSCs can also promote CSCs through IL-6/STAT3 activation and nitric oxide/Notch cross-talk signaling. Notch helps sustain STAT3 signaling which is important for CSC maintenance. (iv) Similar to MDSCs, regulatory T cells (Tregs) also promote evasion of immune surveillance. Notch-1–TGF-β signaling cooperatively regulates Foxp3 gene expression, and hence directly induces peripheral Tregs. (v) On the other hand, DLL-1 expressing dendritic cells or stromal cells can activate Notch in cytolytic T cells and enhance antitumor activities. (vi) Moreover, endothelial cells contribute to tumor progression and metastasis. Notch1 controls macrophage recruitment to endothelial cells and facilitate vessel branching, which can increase metastasis. (vii) In addition to facilitating the invasion of cancer cells, endothelial cells play a role as CSC niches by releasing supportive factors or by direct cellular contact.

It is now well appreciated that inflammatory responses play key roles at different stages of tumor development, from initiation to malignant conversion, invasion, and metastasis, as well as therapy resistance and recurrence (44). Depending on its type, tumor-induced inflammation consists of innate immune cells including macrophages, neutrophils, mast cells, myeloid-derived suppressor cells (MDSCs), DCs, and NK cells and adaptive immune cells such T cells (effector cells—Th cells and Tregs—and NKT cells) and B cells (44). The interplay between tumor cells and immune cells in the tumor microenvironment dictates the overall immune surveillance and responses to therapies, and subsequently clinical outcome and patient survival. Notch regulates many components of the tumor microenvironment including immune cells as well as fibroblasts, endothelial, and mesenchymal cells (Figure 2) (42, 43, 45, 46).

### Tumor Initiation and Cancer Stem Cells (CSCs)

Immune cells contribute to and enhance tumor initiation and progression through various mechanisms including activating chronic inflammation and tissue repair, angiogenesis, and the induction of pre-malignant cell proliferation, and/or CSCs. CSCs or tumor-initiating cells are a subpopulation of cancer cells that mediate primary tumor formation and metastasis, as well as resistance to therapies through self-renewal activities and immune evasion. Moreover, they are associated with cancer progression, resistance and recurrence, and clinical outcome in cancer patients (47). Elevated Notch pathway activity has been detected in the CSC subpopulation of many cancer types including medulloblastoma, breast, intestine, prostate, and colon cancer, pancreatic ductal adenocarcinoma (PDAC), and squamous cell carcinoma (47–52). The role of Notch in breast cancer and breast CSCs is very well studied. Notch plays a crucial role both in initiation and progression of breast cancer (53). Both Notch1 and Notch4 are found to have differential activities in breast cancer cell lines and patient samples, with Notch4 being the major receptor in the CSC populations of luminal and basal breast cancer cell lines (54, 55). Notch4 and Notch3 are expressed at higher levels in poorly differentiated basal breast cancers and are associated with poor overall survival (54–56). By using a Notch antagonist-γ-secretase (GSI), CSC populations were decreased *in vivo*. An additive effect was detected with GSI and Docetaxel, suggesting that combination therapies with Notch targeted therapies might be beneficial in treating heterogeneous cancer cell populations (55). Moreover, early-phase clinical trials of GSI in breast cancer have provided a limited clinical benefit which can be explained by its activity against CSCs (57). Both Jagged-1 and Jagged-2 have been shown to regulate Notch signaling in breast cancer (56, 58). High expression of Jagged-1 has been detected in aggressive tumors especially triple-negative breast cancer (TNBC) and associated with increased tumor relapse, drug resistance, and metastasis (53). Several studies have shown that Jagged-1 is elevated in endocrine-resistant luminal breast cancers leading to an increase in CSC activity (59). Jagged-2 is also upregulated by hypoxia and results in increased CSCs (60).

Besides the cell intrinsic effects, Jagged-1 expression induced by endocrine therapy resistance affects the tumor microenvironment by induction of macrophage differentiation toward tumor-associated macrophages (TAMs) (61). TAMs are the most frequently found immune cells within the tumor microenvironment that play an important role in suppressing immune surveillance (44). TAMs acquire an anti-inflammatory phenotype, which express immunosuppressive cytokines including IL-10 and TGF-β as well as high expression of arginase-1, which promote cell proliferation, tissue remodeling, and angiogenesis (44). By contrast, macrophages, activated by IFNγ and microbial products, secrete pro-inflammatory cytokines including IL-1b, IL-12, IL-6, TNF-α, and inducible nitric oxide synthase, which are capable of killing pathogens and inducing antitumor immune responses (44). In contrast to these results, forced expression of Notch in macrophages can repress TAM activity by upregulating miRNAs including miR-125 and miR148a-3p, and therefore enhance antitumor capacity (62–64). In addition to TAMs, MDSCs promote tumor progression by various mechanisms including suppressing immune cells and enhancing angiogenesis and metastasis (65). MDSCs are immature myeloid cells that in mice are characterized by either having monocytic characteristics M-MDSCs or neutrophilic characteristics polymorphonuclear (PMN)-MDSCs (65). Notch activity (Hes1 expression) is lower in MDSCs, especially in PMN-MDSCs of patients with renal cell carcinoma and in conditioned media from cultures of breast and lung cancer cell lines. This is caused by an inhibitory phosphorylation of the NICD by casein kinase 2, which disrupts the Notch transcriptional complex (66). In addition, inhibition of Notch promotes PMN-MDSCs over M-MDSCs and these cells had less immunosuppressive capacity when compared with the M-MDSCs when using a lower ratio of MDSCs to cancer cells (67). However, another study showed that deregulated Notch activity can cause myelopoiesis and expansion of MDSCs; this was caused by accumulation of a S2-cleaved Notch receptor, without S3 cleavage, through increased function of ADAM metalloproteases at the S2 site, or inhibition of γ-secretase (Figure 2i) (68). It is not clear if differentiation of M-MDSCs was preferred over PMN-MDSCs in this study and whether the immunosuppressive capacity of MDSCs was affected. These apparently conflicting data suggest that the temporal and special regulation of Notch signaling as well as presence of specific cytokines can impact myeloid differentiation and macrophage polarization during tumor initiation.

We previously have shown that MDSCs are recruited to the tumor microenvironment through activation of the mTOR pathway and production of G-CSF. Furthermore, MDSCs induced Notch signaling in cancer cells and promoted CSC capacity (Figure 2ii) (69). This type of positive feedback loop between cancer cells, immune cells, and CSCs has been observed previously. MDSCs can also promote CSCs through IL-6/STAT3 activation and nitric oxide/Notch cross-talk signaling. Notch helps sustain STAT3 signaling (Figure 2iii) (70). IL-6-STAT3 activation also results in both the expansion of MDSCs and their circulation in various cancer types (71). Moreover, cancer cells increase Jagged-1 and Jagged-2 expression in MDSCs through NFκB-P65 signaling which results in tumor-induced T cell tolerance (72). The presence of MDSCs (CD33 staining and a G-CSF gene signature) correlates with CSC properties in clinical specimens and predicts poor survival outcome (69, 70). Therefore, targeting MDSCs can be beneficial both by decreasing immunosuppression and inhibiting CSCs.

Similar to MDSCs, Tregs also promote evasion of immune surveillance and are associated with tumor invasiveness and poor clinical outcome. Notch1, which is a key regulator of luminal estrogen receptor (ER^+^) breast cancers is inversely correlated with the aggressive TNBC/basal-like breast carcinomas and infiltrating Foxp3^+^ Tregs (73). However, Notch-1–TGF-β signaling cooperatively regulates Foxp3 gene expression, and hence directly induces peripheral Tregs (Figure 2iv) (32). Given that Notch4 and Notch3 are expressed at higher levels in poorly differentiated basal breast cancers (54–56) it will be important to elucidate the association of different Notch pathways with Tregs during cancer formation. Moreover, it will be important to understand the regulation of different Notch ligands in different cancer types and the recruitment of Tregs. Both Jagged-1 and Jagged-2 increase the generation of Tregs (33) and both of these ligands are highly expressed in TNBC, CSCs, and the treatment-resistant populations (53, 59, 60). Interestingly, in an experimental model of autoimmune diabetes, it was demonstrated that Notch3 expression in the lymphoid organs results in generation of Tregs. These Tregs secrete suppressive cytokines such as IL-4 and IL-10 and express cytotoxic T lymphocyte-associated protein 4 (CTLA-4) (74), a receptor shown to block T cell co-stimulation, by competing with CD28 for B7 ligand, and therefore abrogate an activated T cell response (75).

Notch also regulates the antitumor immune response. Increased T cell numbers, specifically activated CD8^+^ CTLs and Th1 cells, correlate with better survival in many cancers (44). Both Notch1 and Notch2 were shown to directly regulate CTL-specific gene expression including granzyme B (35, 36). Notch2, but not Notch1-deficient CD8^+^ T cells were unable to expand and suppress tumors in mice (76). In addition, Notch2 agonists or DLL-1 expressing DCs or stromal cells enhanced CTL activity and eradicated tumors (76, 77). Using a mouse model of lung cancer, systemic administration of multivalent forms of DLL-1 enhanced the Th1 response through STAT1/STAT2/T-bet resulting in an increase in T cell infiltration into tumors and CD8^+^ memory cells, as well as a decrease in Tregs, and tumor vascularization (78). In addition, progression-free survival was increased when the multivalent DLL-1 was combined with EGFR-targeted therapy, Erlotinib, as a result of augmented tumor-induced T cell immunity (78). The soluble clustered DLL1 acts as an activator of Notch receptors, whereas soluble forms of DLL1 (or other Notch ligands) act as inhibitors of Notch signaling (77). In a recent study, a role for Notch in generating antigen-specific stem cell memory T (T_SCM_) for adoptive immunotherapy cells has been described. T_SCM_ cells were generated from activated CD4^+^ and CD8^+^ T cells by co-culturing with stromal cells that expressed DLL1 (79). These long-lived and highly proliferative memory T cells were shown to lose the markers for exhausted T cells, programmed cell death protein 1 (PD-1), and CTLA-4 and to elicit antitumor activities (Figure 2v) (79).

A subset of DCs with high DLL4 expression has been described recently. DLL4^+^ DCs were essential for an effective antitumor response. Under low doses of antigen, DLL4-Notch signaling acts as a co-stimulator to potentiate phosphatidylinositol 3-OH kinase (PI3K)-dependent signaling downstream of the TCR-CD28, and therefore enhances CD4^+^ T cell to elicit an effective antitumor response (80). This subset of DLL4^+^ DCs has also been found in human peripheral blood under inflammatory conditions and was shown to be more efficient in promoting Th1 and Th17 differentiation (40). However, its role in cancer patients has not yet been studied. This is very important because there are now several blocking antibodies against DLL4 being tested in clinical trials. Additional evidence for the essential role of Notch in regulating DC-dependent antitumor immune response comes from a study where RBP-J-deficient DCs were shown to be incapable of inhibiting tumor growth due to their decreased capacity to activate and/or recruit T, B, and NK cells (81). Therefore, it is important to understand which specific combination of Notch ligands and receptors contribute to the heterogeneous population of tumor and tumor microenvironment.

### Angiogenesis and Metastasis

Tumor progression and the initiation of invasion and metastasis are supported by angiogenesis. DLL4–Notch1 signaling was shown to coordinate the formation of the endothelial “tip cells” in relation to the “stalk cells” required for the correct sprouting and branching patterns during angiogenesis (82). Notch1 controls macrophage recruitment during retinal angiogenesis in mice and these macrophages interact with the DLL4-positive tip cells to facilitate the bridging between sprouts or vessel anastomosis (Figure 2vi) (42). Endothelial cells are suggested to play a role as CSC niches by releasing supportive factors or by direct cellular contact (Figure 2vii) (83). Endothelial cells were shown to support glioblastoma multiforme (GBM) CSCs by providing Notch ligands. Furthermore, Notch inhibition in endothelial cells blocked self-renewal of the CSCs and GBM tumor growth (84). In ovarian cancer, Notch (Jagged-1 expression) enhances tumor progression by supporting both cancer cell proliferation, chemoresistance, and endothelial cell regulating angiogenesis (45, 46).

Besides supporting CSCs, endothelial cells regulate the passage of cancer cells and immune cells across the endothelium lumen. Notch signaling is implicated in promoting inflamed endothelium which results in opening of gap junctions and promoting the adhesion of tumor cells (85, 86). This enhances migration of leukocytes, and potentially cancer cells, across endothelium. A recent study has shown that endothelial Notch1 can be activated by tumor cells and myeloid cells at a distant metastatic site (lung) (87). Sustained Notch activation induced inflamed endothelium which expressed the adhesion molecule VCAM1; this further promoted neutrophil infiltration, tumor cell adhesion to the endothelium, and intravasation at the primary site, as well as extravasation to the pre-metastatic niche (87).

Notch is also implicated in regulating the epithelial–mesenchymal transition (EMT) in various cancers including breast, prostate, pancreatic, and squamous cell carcinoma (49, 88–90). In both breast and pancreatic cancers, Jagged-1 expression is associated with EMT including increased Slug gene expression, and inhibiting Notch decreased metastasis (88, 89). In particular, gemcitabine-resistant pancreatic cancer acquired EMT properties and a CSC phenotype through Jagged1–Notch2 (89), suggesting that inactivation of Notch may be a potential therapeutic approach to overcome chemoresistance in invasive and metastatic pancreatic cancer. In another study, Jagged-1 expression was correlated with high tumor grade and vascular invasion, and shorter disease-free survival in breast cancer (91). Elevated Jagged-1 expression also correlates with positive lymph node, metastatic relapse, and a higher number of disseminated tumor cells in bone marrow aspirates (91). Interestingly, in patients with detectable circulating tumor cells (CTCs), more than 85% of CTCs express Jagged-1 (91), suggesting that Notch may be implicated in the survival of disseminated tumor cells and metastasis.

At the metastatic site, tumor-derived Jagged-1 promotes osteolytic bone metastasis in breast cancer (92). Notch activation in osteoblasts induces the expression and secretion of IL-6, which in return supports the growth of tumor cells (92). Meanwhile, in osteoclasts (bone-resorbing cells differentiated from monocyte/macrophage precursors), Notch directly controls the maturation of these cells, and therefore enhances osteolytic function (92). Release of TGF-β as a result of bone destruction triggers a positive feedback loop to sustain Jagged-1 expression in tumor cells and therefore maintains the osteolytic environment (92).

### Drug Resistance, Dormancy, and Recurrence

Notch is implicated in drug resistance and survival of dormant cells. Elevated Notch signaling is associated with therapeutic resistance and increased risk for tumor recurrence in breast cancer patients (93). Using a Her2/neu mouse model of mammary gland tumors, Notch signaling was shown to be activated in a subset of dormant residual cells following anti-Her2 therapies. Furthermore, Notch accelerated tumor recurrence (93). Another study showed that ErbB-2 inhibition by a monoclonal antibody Trastuzumab activated Notch1 in breast cancer cell lines and Trastuzumab-resistant cells showed higher Notch activity (94). In both studies inhibiting Notch impaired tumor recurrence (93, 94). Moreover, Notch signaling has been implicated in endocrine-resistant breast cancer (59, 95). Jagged-1–Notch4 is highly expressed in resistant CSCs resulted from anti-estrogen therapy and combining endocrine therapy with Notch inhibition overcame this resistance (59). The presence of TAMs in the microenvironment correlates with tamoxifen resistance and decreased survival of postmenopausal breast cancer patients (96). Jagged-1 upregulation in endocrine-resistant breast cancer modulates the differentiation and polarization of macrophages to TAMs to promote the metastatic potential of cancer cells (61). Notch activation has also been implicated in resistance against chemotherapy by either inducing a CSC phenotype or promoting intratumoral heterogeneity (90, 97, 98), thus suggesting that combination therapies may be more efficacious.

## Therapeutic Targeting of Notch

The extensive study of Notch pathway regulation has provided us with many potential avenues for Notch modulation including inhibiting ligand–receptor interactions or proteolytic activation of the receptor. GSIs are the best studied small molecules targeting the Notch pathway. GSIs prevent Notch from being cleaved and reduce the levels of intracellular activated Notch (53). There are several GSIs at different stages of clinical trials, including MK-0752 and RO4929097. In preclinical studies, the MK-0752 inhibitor—MRK-003—decreased CSCs in breast cancer and PDAC models (99, 100). Using various other GSIs, including RO4929097, GBM CSCs also were significantly decreased (101). Moreover, about 45% of GBM patients had high Notch pathway activity and were predicted to respond to GSIs (101). GSI monotherapies are associated with gastrointestinal toxicities, but in combination with chemotherapy and glucocorticoids, they can be both more efficacious and less toxic (102). In fact, MK-0752 treatment improved the activity of docetaxel and reduced breast CSCs (57). Moreover, a clinical trial of RO4929097 with chemotherapy (paclitaxel and carboplatin) showed complete pathologic response in 50% of TNBC patients (53). Additional details about different GSI clinical trials have been reviewed elsewhere (103).

Therapeutic antibodies may demonstrate better efficacy and specificity than small molecule inhibitors in cancer therapy. Several blocking antibodies against DLL4 are being tested in phase I clinical trials. Demcizumab (OMP-21M18) may inhibit CSCs and angiogenesis and is being tested in various cancer types including non-small-cell lung cancer, ovarian, and pancreatic cancer (103). Results from phase Ib trial of Demcizumab in combination with chemotherapy showed some clinical benefit, however, it did not meet the expected endpoints (47, 53). Rovalpituzumab tesirine (SC16LD6.5) is an antibody–drug conjugate consisting of DLL3-specific IgG1 monoclonal antibody SC16 and the DNA cross-linking agent SC-DR002 (D6.5) (104). Rovalpituzumab tesirine exhibited encouraging single-agent antitumor activity in small-cell lung cancer patients who express high levels of DLL3 (104).

Antibodies have been developed to target Notch1, Notch2, or Notch3. Tarexumab (OMP-59R5) is an antibody against Notch 2 and Notch3 that can inhibit CSCs and tumor growth (47, 105). Tarexumab is being tested in phase II trials for the treatment of pancreatic cancer and small-cell lung cancer (103). Brontictuzumab (OMP-52M51), an anti-Notch1 antibody, has shown to reduce CTCs and provided some efficacy in patients with metastatic colorectal cancer (103).

## Conclusion and Future Direction

Most of the Notch therapeutics have been tolerated by patients. Although they are associated with various adverse effects, these effects are usually manageable. However, the effects of these therapies on tumor immunology are not well studied. Immunotherapies are revolutionizing the treatment of many cancers. Inhibiting negative regulators of immune activation (immune checkpoint) through immune checkpoint blockade therapies (ICBT) has been remarkably effective in several cancer types including metastatic melanoma and non-small cell lung cancer (106, 107). These treatments target negative regulators of T cell activity, thereby unleashing antitumor immunity. Two very successful strategies of ICBT have been achieved by antibodies blocking the CTLA-4 or the PD-1 pathways, either alone or in combination (108). Therefore, it is necessary to understand the effect of Notch therapeutics on tumor immunology. As discussed earlier, both *in vitro* and *in vivo* studies have associated DLL family ligands with the development of IFNγ-secreting Th1 cells and Th17, while Jagged family ligands elicit Th2, Th9, and Treg responses (10, 22, 27). Moreover, DLL and Jagged ligands appear to elicit opposite effects in myeloid cells: DLL1 and DLL4 promote differentiation of DCs while activation of Notch through Jagged-1 promotes immature myeloid cells (20). On the other hand, Notch2 controls the survival of DCs (also identified as Cx3cr1^low^ Esam^high^ DC subset), which is required for efficient T cell priming. Therefore, these results suggest that combining anti-Notch2 and DLL therapies with ICBT might not be beneficial because of reduced T cell priming and activation of Th1, Th17, and CD8^+^ T cells that happens through DCs. Moreover, Notch1 and Notch2 have been shown to directly regulate CTL-specific gene expression including granzyme B, therefore prolonging administration of these drugs might suppress CTL activity and again dampen the efficacy of ICBT. On the other hand, anti-Jagged therapies look more promising if combined with ICBT, e.g., in a mouse model, anti-Jegged-1/2 both inhibited MDSCs and induced Notch1 in CD8^+^ T cells, which promoted antitumor T-cell immunity and protective immune memory response (72). The majority of recent studies suggest that because of the broad functions of Notch signaling, we must design better strategies utilizing anti-Notch therapies both by dosage deescalation and by combinations with different therapies as well as designing specific treatment schedules. For example, it may be preferable to use anti-Jagged therapies before ICBT, as the host immune system might be primed to respond better to ICBT or to use even a lower dosage of ICBT and therefore decrease toxicity. Moving forward, there needs to be more research to investigate the effect of Notch therapies on different immune cell compartments and functions to enable the design of combinatorial treatments.

## Author Contributions

MJ and XZ designed the outline of the review and MJ composed the manuscript. LX and JR contributed to the content of and edited the manuscript. XZ edited the manuscript, and supervised the writing process.

## Conflict of Interest Statement

The authors declare that the research was conducted in the absence of any commercial or financial relationships that could be construed as a potential conflict of interest.
